# Effects of a Combination of Extracts from Olive Fruit and Almonds Skin on Oxidative and Inflammation Markers in Hypercholesterolemic Subjects: A Randomized Controlled Trial

**DOI:** 10.1089/jmf.2020.0088

**Published:** 2021-05-17

**Authors:** Juristo Fonollá, Jose A. Maldonado-Lobón, Roberto Luque, Carlos Rodríguez, Óscar Bañuelos, Jose L. López-Larramendi, Mónica Olivares, Ruth Blanco-Rojo

**Affiliations:** ^1^ Research and Development Department, Biosearch Life, Granada, Spain.; ^2^ Faculty of Pharmacy, University of Granada, Granada, Spain.; ^3^ Commercial and Marketing Department, Biosearch Life, Granada, Spain.

**Keywords:** almond skin polyphenols, atherosclerosis, hydroxytyrosol, inflammation, oxidized low-density lipoproteins

## Abstract

Hydroxytyrosol (HT) from olives and polyphenols from almond skin (ASPs) possess cardioprotective properties. This pilot study evaluates the effect of supplementation with a combination of olive fruit and almond skin extracts on low-density lipoprotein (LDL) cholesterol oxidation, lipid homeostasis, and inflammatory parameters in adults with moderate hypercholesterolemia. A randomized, parallel, double-blind, placebo-controlled pilot study of 8 weeks was performed. The extract group (EG) received the supplement with 7.5 mg HT +210 mg ASPs, and the control group (CG) received a placebo composed of maltodextrin. Oxidized LDL (oxLDL) levels and the oxLDL/LDL ratio were lower in the EG than in the CG after 8 weeks of treatment (18.76 ± 3.91 vs. 10.34 ± 4.22, *P* < .001 and 0.151 ± 0.025 vs. 0.08 ± 0.023, *P* < .001, respectively). Interleukin-1β levels were significantly higher in the CG than in the EG at week 4 (*P* = .004), IL-6 was significantly higher in the CG than in the EG at week 4 (*P* = .049), and IL-10 was significantly increased at week 4 in both groups (*P* = .002 for CG and *P* = .001 for EG). In conclusion, daily consumption of a combination of an olive fruit extract and an almond skin extract for 8 weeks seems to protect LDL from oxidation and to prevent inflammatory status in moderately hypercholesterolemic subjects.

## INTRODUCTION

Cardiovascular diseases (CVD) still are the leading cause of mortality worldwide, being responsible for an estimated 17.8 million deaths in 2017,^1^ which is supposed to be a 12.5% mortality increment since the past decade.^2^

Atherosclerotic vascular disease or atherosclerosis is one of the most important causes of CVD.^3^ This is a chronic vascular inflammatory disease associated to oxidative stress and endothelial dysfunction,^4^ in which oxidized low-density lipoproteins (oxLDLs) have been suggested to play an important role.^5^ OxLDL binds with higher affinity than native LDL to several receptors, so its uptake by macrophages is more rapid, leading to cholesterol accumulation and the formation of atherosclerotic plaque.^6^ Moreover, oxLDL promotes the activation and dysfunction of endothelial cells, the migration and proliferation of vascular smooth muscle cells, and the platelet activation, causing inflammatory response, endothelial dysfunction, and worsening the atherogenic process.^7^ Therefore, circulating level of oxLDL is considered as one of the most important biomarkers in the progression of atherosclerosis.^6^

Nevertheless, it is well known that most of CVD can be prevented by the promotion of a healthy lifestyle,^8^ and several dietary strategies to reduce cardiovascular risk have been developed.^9^ In this regard, the use of polyphenols as nutraceutical agents is spreading,^10,11^ and among those, hydroxytyrosol (HT) from olives and polyphenols from almond skin (ASPs) have been attributed as presenting cardioprotective properties.^12–15^ HT is a phenolic compound present both in the fruit and leaf of the olive (*Olea europaea L*.).^16^ There are several evidences about its anti-inflammatory and anti-atherogenic functions, such as the inhibition of LDL oxidation, platelet aggregation, and other factors involved in the development of atherosclerosis.^17^ Regarding ASPs, the most abundant were proanthocyanidins, hydrolysable tannins, and flavonoids.^18^ Several studies suggested that, beyond the antioxidant and anti-inflammatory properties of these polyphenols, they also improved lipid homeostasis.^13^ Therefore, the combined intake of both bioactive compounds may help to reduce some risk factors associated with CVD.

The aim of this pilot study is to evaluate the effect of supplementation with a combination of an olive fruit extract (standardized in HT) and an almond skin extract (standardized in total polyphenols) on LDL-cholesterol (LDL-c) oxidation in adults with moderately hypercholesterolemia. As secondary outcomes, lipid homeostasis and inflammatory parameters are also evaluated.

## MATERIALS AND METHODS

### Study subjects

Volunteers were recruited from the Endocrinology Service of Hospital San Cecilio in Granada (Spain) and from the database of the Clinical Studies Unit of Biosearch Life. Study participants met the following inclusion criteria: men and women from 18 to 65 years old that have moderately hypercholesterolemia (LDL-c >100 mg/dL and/or total cholesterol (TC) >200 mg/dL) and that are not receiving medical treatment for this condition. Exclusion criteria included pregnancy, to have a major medical problem, diabetes and/or neurovascular disease, and to present allergy to antibiotics or to some of the ingredients of the study. Subjects were also excluded if they were on diet or they were taking food supplements during the study. The study was conducted according to the Declaration of Helsinki, and the protocol was approved by the Regional Ethics Committee (Granada, Spain). Informed consent was obtained from all subjects. The trial was registered in the U.S. Library of Medicine (www.clinicaltrial.gov) as NCT04029727.

**FIG. 1.**
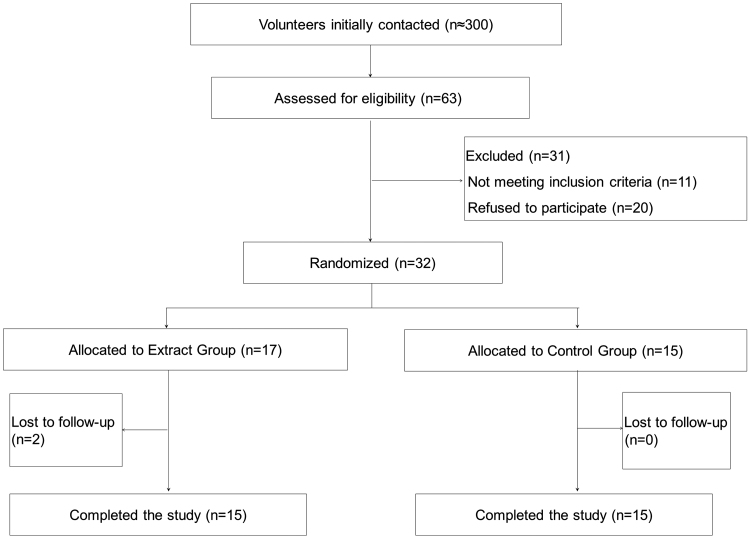
Diagram of the consolidated standards of reporting trials.

Approximately 300 subjects were contacted and invited to participate in the study, and 63 were assessed for eligibility. Out of them, 31 were excluded (11 did not meet the inclusion criteria, and the rest refused to participate). Finally, a total of 32 subjects agreed to participate in the intervention and were randomized (Fig. 1).

### Study design

This was a randomized, parallel, double-blind, placebo-controlled pilot study of 8 weeks of duration. The study was performed between May and July 2019. A group of 32 subjects were enrolled and randomly assigned into two different groups according to a randomization scheme generated by a computer program (SIGESMU^®^). One group (extract group [EG]) received an oral supplementation with a combination of olive fruit extract and almond skin extract (7.5 mg HT +210 mg ASPs +33 mg maltodextrin per day), and the other (control group [CG]) received a placebo (800 mg maltodextrin per day). Treatments were given to subjects for 8 weeks, and they consumed two capsules at a time per day (consuming the extract supplement or the placebo) at lunch time. Participating subjects were instructed not to deviate from their regular habits and to maintain their normal diet and exercise level during the 8 weeks. Neither the researchers nor the subjects knew which treatment sequence the subjects had been assigned to; the researchers were unblinded only at the end of the study.

### Study products

The daily dose of the study product consisted on a mix of 67 mg of olive fruit (*Olea europaea L*.) extract standardized to 11% HT and 700 mg of almond skin (*Prunus dulcis (*Mill.)) extract standardized to 30% total polyphenols. Therefore, volunteers consumed 7.5 mg HT +210 mg ASPs per day. Dose of HT was based on European Food Safety Authority (EFSA) claim for HT consumption and LDL protection from oxidation^19^ and the differences observed in HT bioavailability among several matrices.^20^ Dose of ASPs was selected for being the most effective in avoiding the degradation of the HT during digestion.^21^ Since volunteers consumed two capsules per day, each extract capsule contained 3.75 mg of HT from 33.5 mg of the standardized olive fruit extract, 105 mg of ASPs from 350 mg of the standardized almond skin extract, and 16.5 mg of maltodextrin. Each placebo capsule contained 400 mg of maltodextrin. Both extracts were obtained in the production plant of Biosearch Life in Talayuela, Cáceres (Spain), and the combination of the extracts was performed in the R&D facilities of Biosearch Life in Granada (Spain). Extracts were obtained from the fruit of *O. europaea* and the skin of *P. dulcis* through standardized extraction processes described elsewhere.^21^ The content of HT was analyzed by high-performance liquid chromatography with a diode-array detector (HPLC-DAD). HT maximum peak was detected at 280 nm with a retention time of 10.5 min.^21^ The content of total ASPs was determined by the Folin–Ciocalteu method.^21^ Capsules were prepared in the Department of Pharmaceutical Technology of the Faculty of Pharmacy of the University of Granada (Spain). The extracts and the placebo were provided in identical gelatin capsules packaged in identical plastic containers with a code number that referred to the volunteer code according to the randomization.

### Study outcomes and data collection

The primary outcome of the study was the determination of oxLDL levels. Secondary outcomes included oxLDL/LDL-c ratio, TC, LDL-c levels, high-density lipoprotein-cholesterol (HDL-c) and serum triglycerides (TG) levels, and serum levels of the cytokines interleukin-1β (IL-1β), IL-6, and IL-10.

Subjects attended to Biosearch Life facilities in Granada at baseline, 4 weeks, and 8 weeks. Blood samples were collected after an overnight fast lasting at least 10 hours at baseline and at every follow-up visit, using Vacutainer^®^ SST™ II Advance Tubes (BD, NJ) containing a thixotropic gel. Serum was obtained after centrifugation at 1000 *g* for 15 minutes and stored at −80°C.

Anthropometric measures were taken at every visit using standardized methods. Weight was determined using the Tanita BC-418 Body Composition Analyzer (Tanita, Tokyo, Japan). Height was determined using a height meter with an accuracy of 1 mm (range, 80–200 cm). Body mass index (BMI) was calculated as weight/height squared (kg/m^2^). All measurements were made by trained personnel.

Each subject's dietary intake was evaluated at baseline and at the end of the study to control any possible changes in their habitual habits. Subjects completed a 72 hours detailed dietary intake report, specifying the types of food consumed and serving weights. Daily food, energy intake, nutrient intake, and energy provided by macronutrients were calculated by the computer application Nutriber (Funiber, Barcelona, Spain). Subjects were also asked about their physical activity habits (type of activity and time performing per week) at baseline and at the end of the study.

Compliance was assessed at the end of the intervention by comparing the number of capsules provided and the number returned. Adverse events, defined as any unfavorable unintended effect, were recorded on the follow-up visits (at 4 and 8 weeks).

### Biochemical parameter analysis

Lipid parameter levels (TC, LDL-c, HDL-c, and TG) were analyzed by an external laboratory (Reference Laboratory S.A, Barcelona, Spain) by standardized methods. Determination of oxLDL concentration in serum was done by ELISA Kit that recognizes natural and recombinant human oxLDL (Elabscience Biotechnology). The concentration of OxLDL (pg/mL) of each sample was read from the standard curve and finally expressed in ng/dl. Then the oxLDL/LDL-c ratio was calculated in ng/mg. Serum levels of IL-1β, IL-6, and IL-10 were analyzed by human uncoated ELISA Kits (Thermo Fisher Scientific).

### Statistical analysis

Normal distribution was tested for all measured variables by normal probability plots and the Shapiro–Wilk test. Data are presented as mean (standard deviation) for continuous variables and as *n* (%) for categorical variables.

For comparisons between groups at the beginning of the study (extract vs. control), the continuous variables were analyzed with the Student *t*-test or the nonparametric method of Kruskal–Wallis, as appropriate, and categorical variables were analyzed with the chi-square tests.

All parameters will be compared between the groups, and the change with respect to the initial values per group will also be analyzed. For this purpose, bivariate analyses will be carried out, as well as the adjusted test based on the mixed regression model. The comparison between the means of the experimental group and the CG shall be carried out by means of the *t*-test when normality can be assumed. When non-normality can be assumed, nonparametric Mann–Whitney *U* test was performed. In addition, the mixed linear regression model that represents repeated measurements throughout the study (intrasubject-random effect) will be adjusted to the responses to test for change over time and between groups, as well as to find significant factors associated with the responses.

A general alpha level of 0.05 will be used as the cutoff point for statistical significance. Statistical analysis was carried out using SPSS software version 26.0 for Windows (SPSS, Chicago, IL).

## RESULTS

A total of 30 subjects completed the study, since two subjects in the EG refused to participate before attending the baseline visit (Fig. 1). Compliance rate was confirmed to be very high (∼100%). No adverse events resulting from the intake of either type of treatment capsule were reported.

Of the 30 volunteers that finished the study, 13 were men (43.43%) and 17 were women (56.7%). Mean age of the subjects was 48.13 ± 12.9 years old (age range 20–64), and they presented a mean BMI of 25.38 ± 3.33 kg/m^2^. No significant differences were detected in the baseline characteristics of the volunteers except in the levels of cytokines IL-6, which was significantly higher in the CG (Table 1).

**Table 1.** Baseline Characteristics of the Subjects Participating in the Study

	CG (*n* = 15)	EG (*n* = 15)	P-between groups
Age (years)	50.26 ± 13.29	46 ± 12.67	.376
Sex			.713
Males	6 (40)	7 (46.7)	
Females	9 (60)	8 (53.3)	
BMI (kg/m^2^)	25.57 ± 3.69	25.20 ± 3.05	.769
Obesity or overweight (>25 kg/m^2^)	9 (60)	8 (53.3)	.713
Smokers	2 (13.3)	2 (13.3)	1.000
Practice physical activity regularly	10 (66.7)	8 (53.3)	.456
Total-cholesterol (mg/dL)	228.26 ± 28.09	222.93 ± 22.33	.570
LDL-c (mg/dL)	120.26 ± 24.04	124.33 ± 20.34	.621
HDL-c (mg/dL)	65.8 ± 12.49	60.05 ± 16.87	.298
Serum triglycerides (mg/dL)	95 ± 34.98	101.93 ± 44	.637
Oxidized-LDL (ng/dL)	12.34 ± 6.13	12.01 ± 6.38	.887
Oxidized-LDL/LDL-c ratio (ng/mg)	0.104 ± 0.055	0.103 ± 0.058	.952
IL-1β (pg/mL)	0.55 ± 0.25	0.32 ± 0.37	.063
IL-6 (pg/mL)	0.58 ± 0.24	0.33 ± 0.18	.004
IL-10 (pg/mL)	0.26 ± 0.33	0.06 ± 0.32	.096

Values are mean ± SD for continuous variables and *n* (%) for categorical variables. *P* indicates differences between groups.

BMI, body mass index; CG, control group; EG, extract group; HDL-c, high-density lipoprotein-cholesterol; IL-1β, interleukin-1β; LDL, low-density lipoprotein; LDL-c, LDL-cholesterol; SD, standard deviation.

Energy and nutrient intakes at baseline and week 8 are shown in Table 2. There were no significant differences at baseline between the intervention groups nor between baseline and the end of the intervention in each group (Extract or Control). There were also no changes during the study in physical activity performance (data not shown), and subjects in both groups neither significantly changed their BMI between baseline and week 8 (Table 2).

**Table 2.** Dietary and Anthropometric Parameters of the Subjects Consuming the Placebo or the Extract During 8 Weeks

	CG (*n* = 15)			EG (*n* = 15)		
	Baseline	8 weeks	P-time	Baseline	8 weeks	P-time
Energy (kcal)	1737.86 ± 277.79	1687.4 ± 281.05	.096	1708.28 ± 471.5	1683.32 ± 386.93	.703
Total fat (%En)	38.53 ± 5.38	35.38 ± 8.81	.287	34.92 ± 5.42	35 ± 5.49	.960
Saturated fat (%En)	8.73 ± 3.27	7.37 ± 2.74	.224	8.93 ± 1.72	8.8 ± 1.84	.863
Monosaturated fat (%En)	18.78 ± 3.77	16.59 ± 4.79	.181	15.94 ± 4.33	16.48 ± 4.13	.637
Polysaturated fat (%En)	5.83 ± 2.59	5.41 ± 2.68	.469	4.35 ± 1.36	4.3 ± 1.63	.878
Dietary cholesterol (mg/day)	218.4 ± 85.44	204.38 ± 88.04	.559	234.72 ± 99	222.76 ± 87.24	.712
Protein (%En)	18.15 ± 2.35	18.55 ± 2.17	.589	20.52 ± 2.47	18.93 ± 5.22	.231
Carbohydrates (%En)	44.19 ± 7.7	45.2 ± 8.46	.726	44.99 ± 7.38	45.47 ± 6.59	.815
Fiber (g/day)	19.45 ± 6.45	16.46 ± 5.94	.073	16.51 ± 5.33	14.73 ± 5.82	.339
BMI (kg/m^2^)	25.57 ± 3.69	25.70 ± 3.87	.524	25.20 ± 3.05	25.31 ± 2.96	.359

Values are mean ± SD. *P* indicates differences between baseline and 8 weeks in each treatment group.

**FIG. 2.**
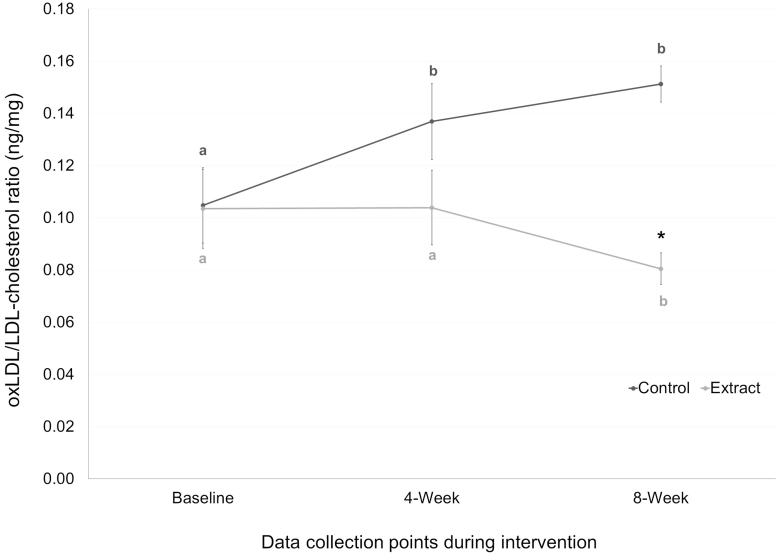
OxLDL/LDL-cholesterol ratio (ng/mg) of the subjects consuming the placebo or the extract at baseline, 4 weeks, and 8 weeks. Mean values are represented with points (*dark gray* for control group and *light gray* for extract group), whereas SE is represented by vertical bars. Different letters mean significant differences between time points in each group. *Asterisk* (*) indicates *P* < .05 between groups at each time point. OxLDL, oxidized low-density lipoprotein; SE, standard error.

Table 3 shows the values for the lipid parameters and the oxidized lipid variables evaluated during the study. No significant changes were observed between baseline and the end of the intervention in TC, LDL-c, HDL-c, or serum TG in any group. Regarding lipid oxidative status, oxLDL levels significantly increased from baseline in the CG at week 4 (*P* = .01) and at week 8 (*P* = .03), whereas tended to decrease in the EG, being significantly lower in the EG than in the CG after 8 weeks of treatment (*P* < .001). When we analyzed the oxLDL/LDL-c ratio, an accurate estimation of *in vivo* LDL oxidation,^22^ we observed a significant increase of the oxLDL/LDL-c ratio from baseline in the CG at week 4 (*P* = .001) and at week 8 (*P* = .002) and a significant decrease of this ratio in the EG at week 8 (*P* = .047), being also significantly lower the oxLDL/LDL-c ratio levels in the EG than in the CG after 8 weeks of treatment (*P* < .001) (Table 3 and Fig. 2).

**Table 3.** Lipid Parameters and Oxidized Low-Density Lipoprotein of the Subjects Consuming the Placebo or the Extract at Baseline, 4 Weeks, and 8 Weeks

	CG	EG	P-between groups
Total cholesterol (mg/dL)			
Baseline	228.26 ± 28.09	222.93 ± 22.33	.570
4 weeks	232.6 ± 36.97	236.06 ± 39.77	.807
8 weeks	232.46 ± 48.39	233.13 ± 26.07	.963
LDL-c (mg/dL)			
Baseline	120.26 ± 24.04	124.33 ± 20.34	.621
4 weeks	128.4 ± 30.85	131.4 ± 27.72	.781
8 weeks	125.46 ± 35.61	131.86 ± 26.23	.580
HDL-c (mg/dL)			
Baseline	65.8 ± 12.49	60.05 ± 16.87	.298
4 weeks	66.39 ± 9.24	57.6 ± 12.87	.073
8 weeks	65.78 ± 14.62	57.53 ± 11.24	.094
Serum triglycerides (mg/dL)			
Baseline	95 ± 34.98	101.93 ± 44	.637
4 weeks	102.6 ± 41.2	106.33 ± 53.43	.832
8 weeks	101.13 ± 39.46	111.4 ± 54.49	.559
OxLDL (ng/dL)			
Baseline	12.34 ± 6.13	12.01 ± 6.38	.887
4 weeks	17.14 ± 6.83^*^	13.24 ± 7.04	.135
8 weeks	18.76 ± 3.91^*^	10.34 ± 4.22	<.001
OxLDL/LDL-c ratio (ng/mg)			
Baseline	0.104 ± 0.055	0.103 ± 0.058	.952
4 weeks	0.136 ± 0.056^*^	0.103 ± 0.055	.117
8 weeks	0.151 ± 0.025^*^	0.08 ± 0.023^*^	<.001

Values are mean ± SD adjusted by sex, age, obesity or overweight, smoking, and physical activity habits. *P* indicates differences between groups at each time point. Asterisks (^*^) indicate *P* < .05 between baseline and 4 weeks and 8 weeks.

OxLDL, oxidized low-density lipoprotein.

Levels of serum cytokines evaluated in this study are shown in Table 4. Levels of IL-1β tended to decrease in both groups at the end of the intervention, although only reached significance at week 8 in the CG (*P* = .014). However, IL-1β levels were significantly higher in the CG than in the EG at week 4 (*P* = .004). Regarding levels of IL-6, they significantly increased at week 4 (*P* = .001) and week 8 (*P* = .002) in the CG, being significantly higher in the CG than in the EG at week 4 (*P* = .049). Levels of IL-10 significantly increased at week 4 in both groups (*P* = .002 for CG and *P* = .001 for EG) and then significantly decreased only in the CG at week 8 (*P* = .026).

**Table 4.** Cytokine Levels of the Subjects Consuming the Placebo or the Extract at Baseline, 4 Weeks, and 8 Weeks

	CG	EG	P-between groups
IL-1β (pg/mL)			
Baseline	0.55 ± 0.25	0.32 ± 0.37	.063
4 weeks	0.58 ± 0.24	0.33 ± 0.18	.004
8 weeks	0.26 ± 0.33^*^	0.06 ± 0.32	.096
IL-6 (pg/mL)			
Baseline	0.23 ± 0.29	0.67 ± 0.51	.007
4 weeks	3.3 ± 2.67^*^	1.56 ± 1.91	.049
8 weeks	3.03 ± 2.7^*^	1.26 ± 2.35	.067
IL-10 (pg/mL)			
Baseline	1.44 ± 1.18	1.54 ± 1.04	.815
4 weeks	2.84 ± 0.7^*^	2.65 ± 1.02^*^	.565
8 weeks	1.31 ± 0.82	0.88 ± 0.73^*^	.137

Values are mean ± SD adjusted by sex, age, obesity or overweight, smoking, and physical activity habits. *P* indicates differences between groups at each time point.

Asterisks (^*^) indicate *P* < .05 between baseline and 4 weeks and 8 weeks.

## DISCUSSION

The present study shows that the daily consumption of 7.5 mg of HT plus 210 mg of ASPs through an oral supplement seems to protect LDL from oxidation and to prevent inflammatory status in subjects with moderately hypercholesterolemia. Several studies have shown the beneficial cardiovascular effect of HT^17^ and ASPs,^18^ but, to our knowledge, this is the first study that evaluated the combined effect of both compounds on cardiovascular risk factor, such as lipid oxidation and inflammation parameters.

There are several clinical trials that reported a decrease in oxLDL levels after the intake of foods rich in HT.^23–25^ Indeed, the EFSA reported the claim that the daily consumption of 5 mg of HT (or its derivatives) from olive protects LDL from oxidative damage.^19^ This polyphenol seems to exert its antioxidant effect by the modulation of different pathways,^26^ such as the activation of multiple genes encoding antioxidant response elements in vascular endothelial cells, the stimulation of mitochondrial biogenesis by increasing *PPARGC1α*, and by the modulation of the expression of miRNAs that may affect the expression of genes involved in atherogenesis.^27^ Regarding ASPs, it has been observed *in vitro* and in animal studies that ASPs may protect LDL against oxidation by acting as ROS scavenger, inducing quinine reductase, and by the stabilization of the LDL conformation.^28–30^ Moreover, a recent randomized acute clinical study reported that milk enriched with ASPs increased the resistance of LDL to oxidation compared to skim milk.^31^ The results obtained in this study support the observed effect of HT and ASPs in protecting LDL from oxidation and, therefore, their role as cardioprotective compounds. Moreover, the combined intake of both ingredients may suppose a synergic action. An *in vitro* study performed by our research group suggests that the addition of ASPs may increase HT bioavailability,^21^ which has been observed to depend on the vehicle used, being more effective when ingested within the olive oil than as an extract.^20^ However, further studies will be needed to test these hypotheses.

Consumption of almonds is proved to have positive effects on lipoprotein profile, due to their favorable fat composition and fiber content.^32^ However, to our knowledge, no study has tested the effect of the almond skin extract on lipoprotein concentration. In addition, the effect of HT on lipid parameter levels remains unclear. Some animal studies showed that HT enriched extracts have reduced plasmatic levels of TC and lipids.^33,34^ However, results in clinical trials are controversial. Covas *et al.*, in a crossover study with healthy men, found no changes in total-cholesterol or in LDL after 3 weeks of intervention with olive oil enriched in HT, but a significant increment of HDL levels.^24^ On the contrary, another study with the same product found no differences in lipid parameter concentration.^25^ Main difference between clinical trial and animal studies is that animals were fed with high fat or high cholesterol diets, and HT attenuated cardiovascular changes associated to these unhealthy diets. Although our volunteers are moderately hypercholesterolemic, they followed a relative healthy and nonchanging diet during the study, so this may be the reason we found no changes in lipid parameters after the intervention.

The anti-inflammatory properties of HT and ASPs have been reported in animal and *in vitro* studies.^35,36^ Moreover, the administration of olive oil enriched in HT decreased inflammatory parameters such as cytokines IL-6 and IL-1β in postprandial^37,38^ and long-term clinical studies.^39,40^ The effect on anti-inflammatory parameters such as the cytokine IL-10 is less clear, although polyphenols from olive may activate this cytokine in carotid plaques from hypertensive patients.^41^ Several authors claimed that oxidative stress and inflammation are inter-related processes, and therefore, the anti-inflammatory effect associated to HT and ASP may be related to the free radical scavenging capacity of both compounds.^42^ Indeed, a postprandial study performed in obese subjects showed that the reduction of the postprandial inflammatory response has attributed to the inhibition of nuclear factor kappa B, which is an important link between oxidation and inflammation in the postprandial state.^38^ In the present study, the intake of the HT+ ASPs supplement avoided the increase of IL-6 compared to the CG and tended to decrease IL-1β values, whereas it activated IL-10 at the midterm of the intervention. These results are in line with those obtained in LDL oxidation levels, taking into account the relationship between oxidation and inflammation. Therefore, through the protection of LDL from oxidative damage, the combined intake of HT and ASPs may prevent the inflammatory status associated to the oxidation process.

One strength of the present study was the design as a double-blind randomized controlled trial controlled by placebo. This methodology allowed to avoid bias related to confounding factors (through a CG), selection bias (through randomization), and interpretation bias (through double blinding). However, a limitation of the study was the low sample size, limiting by the fact that this is a pilot study. In addition, because of the fact that we only recruited moderately hypercholesterolemic subjects, the generalizability of the results cannot be assumed.

A recent study in Spanish population showed that one of the most related traits to the 10-year predicted risk of suffering a coronary event (Framingham-REGICOR score) was oxLDL levels, whereas LDL-c concentration *per se* was not related.^43^ Therefore, therapeutic measures to protect LDL from oxidative damage should be taken, to reduce cardiovascular risk. Our results support the use of the polyphenols HT and ASPs as effective and cardioprotective nutraceutical agents and demonstrated, for the first time, the efficacy of the combined intake of both compounds in protecting LDL from oxidation and preventing inflammation.

In conclusion, the daily consumption of a combination of an olive fruit extract and an almond skin extract (containing 7.5 mg of HT and 210 mg of ASPs) for 8 weeks seems to protect LDL from oxidation and to prevent inflammatory status in subjects with moderately hypercholesterolemia. This, together with the fact that we are using ingredients from foods, and the study subjects not having suffered from adverse effects make the use of this combination of extracts from olive fruit and almond skin, rich in HT and ASPs, a suitable and secure oral supplement that helps to reduce cardiovascular risk in moderately hypercholesterolemic subjects.

## AUTHORS' CONTRIBUTIONS

J.L.L-L., M.O., and R.B-R. were responsible for study concept and design, J.F., J.A.M.-L., R.L., C.R., and O.B were responsible for carrying out the trial and data handling/validation. R.B-R. analyzed and interpreted the data and drafted the article, and M.O. reviewed the article and supervised the project. All authors have read and agreed to the published version of the article.

## ETHICAL APPROVAL

This study was designed and conducted in accordance with the ethical standards as put forth in the Declaration of Helsinki 1975, as revised in 2008. The study protocol, informed consent forms, and associated documents were reviewed and approved by the Regional Ethics Committee (Granada, Spain). The trial was registered in the U.S. Library of Medicine (www.clinicaltrial.gov) as NCT04029727.
